# Midterm results after treatment of gram-positive deep sternal wound infections with daptomycin for cardiac surgery patients

**DOI:** 10.1186/1749-8090-8-21

**Published:** 2013-01-26

**Authors:** Katharina R Ort, Fawad A Jebran, Christian Bireta, Bernhard C Danner, Ioannis Bougioukas, Friedrich A Schoendube, Aron F Popov

**Affiliations:** 1 Department of Thoracic Cardiovascular Surgery, University of Göttingen, Robert-Koch-Strasse 40, Göttingen, 37099, Germany

**Keywords:** Cardiac surgery, Deep sternal infection, Antibiotic treatment, Daptomycin, Midterm outcome

## Abstract

Daptomycin in combination with surgical therapy has shown to be effective for treatment of deep sternal wound infection in cardiac surgery. However, till now midterm results in terms of re-infection or re-operation in patients who were successfully treated with daptomycin for gram-positive deep sternal wound infection are not published. Herein, we present midterm results in patients treated successfully with daptomycin after cardiac surgery.

## Findings

### Methods

This follow up study includes 23 patients. These patients were treated in the past with daptomycin combined with multiple surgeries for deep sternal wound infection (DSWI) due to gram-positive organisms. The initial surveillance was achieved in 100% and wound healing was successfully established in all patients at the time of discharge for the initial clinical course [1]. After discharge, all patients were followed up in terms of mortality, re-infection, and re-operation.

## Results

None of the patients were lost to follow up and follow-up time ranged from 3–42 months (mean 26 months, cumulative 598 patient-months). Four patients (17%) died during the follow up. Three of them died within 3 months of discharge; one due to mitral valve endocarditis, one due to urosepsis, and cause of death could not be established in one. The last patient died after 24 months and cause of death could not be established in him as well. No documented wound complications occurred in any of these patients from the time of discharge till their death.

Two patients (8.5%) after discharge developed superficial wound complications, which were treated surgically. The first patient had a coronary artery bypass grafting and postoperatively developed a DSWI with a *Staphylococcus aureus* and was discharged after 4 weeks in a good clinical condition*.* Thirty-two months after discharge, he developed superficial wound dehiscence, however without any microbial infection.

**Figure 1:**
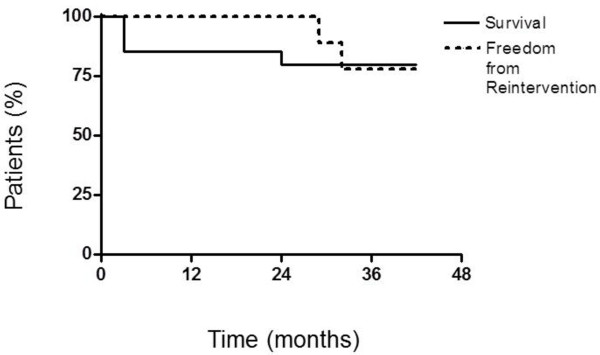
Survival and Freedom from Reintervention.

Other patient had a coronary artery bypass grafting combined with aortic valve replacement. Postoperatively, he developed a DSWI with *Staphylococcus aureus* methicillin susceptible, and after treatment with daptomycin and negative pressure wound therapy (NPWT), a complete wound healing was achieved. Twenty-nine months after discharge, the patient was presented again with a superficial wound dehiscence. The wound culture was positive for *Staphylococcus aureus* methicillin susceptible and the patient was colonized completely. Following multiple surgeries, decolonization and NPWT the patient was discharged home in a good clinical condition. The survival and the requirement for re-operation are summarized in Figure 1.

### Comment

DSWI after cardiac surgery with sternotomy is still a serious surgical challenge. Several treatment options are described in the literature to deal with this complication. A wide range of strategies have been proposed for the treatment of DSWI, including an intense course of directed antibiotic therapy together with a series of debridements and multiple dressing changes [2]. Interestingly, data on the optimal antibiotic management or duration of therapy for DSWI is scarce. Daptomycin has shown to be effective combined with multiple surgeries for treatment of DSWI in cardiac surgery [1,3,4]. However, up till now, no study has shown midterm results in terms of re-infection or re-operation for patients who were successfully treated with daptomycin for gram-positive DSWI.

We have published our results of prospective study about treatment of gram-positive DSWI with daptomycin for cardiac surgery patients performed between February 2009 and September 2010 [1]. Twenty-three consecutive patients with post-sternotomy mediastinitis from gram-positive organisms (out of 1574 primary sternotomies) were identified and treated with intravenous daptomycin with acceptable mortality and morbidity. However, the major drawback of our study was limited observation period lacking any information regarding post-discharge re-infection or re-operation.

Herein, we present our results of discharged patient’s outcome which were treated successfully with daptomycin after cardiac surgery in a midterm follow up.

We had no recurrence of DSWI, which is observed occasionally after cardiac surgery [5]. However, we had one case with superficial wound infection with *Staphylococcus aureus* methicillin susceptible which was treated successfully again with daptomycin.

Also, the mortality in the follow up in patients with DSWI was not different compared to contemporary study [5]. This follow up study confirms that daptomycin can be considered as a viable treatment option for surgical management of gram-positive DSWI after cardiac surgery combined with surgical therapy. Our series presents several limitations; mainly lack of randomization and a small cohort. Further studies are warranted to confirm our single centre experience with daptomycin in treatment of gram-positive DSWI.

## Competing interests

The authors report no competing interests.

## Authors’ contributions

AFP and KO designed the study; FAJ, CB and IB were involved in collecting data; BCD and FAS gave critical comments on the results. All authors read and approved the final manuscript.
